# Correlation between microvessel maturity and ISUP grades assessed using contrast-enhanced transrectal ultrasonography in prostate cancer

**DOI:** 10.1515/med-2023-0772

**Published:** 2023-08-09

**Authors:** Yong Gao, Xuerong Zeng, Xinhong Liao

**Affiliations:** Department of Ultrasound, First Affiliated Hospital of Guangxi Medical University, 530021 Guangxi, China; Department of Ultrasound, First Affiliated Hospital of Guangxi Medical University, 6 Shuangyong Rd, Nanning, 530021 Guangxi, China

**Keywords:** microvessel maturity, MVD, contrast-enhanced transrectal ultrasonography, prostate cancer, ISUP

## Abstract

This study aimed to assess the correlation among the peak intensity (PI) values of quantitative parameters, microvessel density (MVD), microvessel maturity, and International Society of Urological Pathology (ISUP) grades in biopsy specimens from prostate cancer (PCa) patients. The study population included PCa patients who underwent targeted and systematic biopsy, without radiation or chemohormonal therapy before biopsy. Contrast-enhanced transrectal ultrasonography (CE-TRUS) was performed in all patients before biopsy. Contrast-enhancement patterns and PI values of quantitative parameters were observed. Tumor tissue samples were immunostained for CD31 expression. MVD, microvessel maturity, and ISUP grades were determined in prostate biopsy specimens. Based on the contrast enhancement patterns of prostate lesions, 16 patients were assigned to a low-enhancement group and 45 to a high-enhancement group. The number of mature vessels, MVD, mature vessel index, and ISUP grades were all higher in the high-enhancement group than in the low-enhancement group (all *P* < 0.05). The immature vessel index was lower in the high-enhancement group than in the low-enhancement group (*P* < 0.05). The PI value was positively correlated with the number of mature vessels (*r* = 0.372). In conclusion, enhancement patterns on CE-TRUS can reflect microvessel maturity in PCa. The PI value was positively correlated with the number of mature vessels.

## 1 Introduction

The incidence of prostate cancer (PCa) has increased rapidly in recent years around the globe [1]. PCa is a biologically heterogeneous disease. With a timely diagnosis of PCa (recurrent or primary), curative treatment can be provided to patients. Moreover, systemic treatment for localized advanced PCa can offer promising results in terms of disease control and improvement in quality of life [2]. However, some patients with organ-confined PCa eventually develop metastases [3]. In 2014, the International Society of Urological Pathology (ISUP) held a consensus conference that proposed the replacement of Gleason scores with five grades of PCa [4]. ISUP grade is more accurate in predicting disease progression. Grade 1 is the lowest ISUP grade, in which the survival rate without biochemical recurrence is 94.6% [5]. However, Grade 5 is the highest ISUP grade, in which the survival rate without biochemical recurrence is 34.5%. A higher ISUP grade may indicate more aggressive PCa with the worst prognosis [6].

Angiogenesis in PCa is one of the potential pathways promoting the heterogeneity of the disease. Studies have shown that microvessel density (MVD) is closely related to the progression and metastasis of PCa [7]. In previous studies, researchers have used different imaging techniques, such as ultrasonography, positron emission tomography, magnetic resonance imaging, and computed tomography, to evaluate MVD by direct measurement of single or combined parameters [8,9]. Currently, contrast-enhanced transrectal ultrasonography (CE-TRUS) is being used for this purpose. Lee et al. established that the correlation between the maximum intensity of contrast-enhanced ultrasound (CEUS) and the count of CD31-positive microvessels is statistically significant [10]. Jiang et al. suggested a statistically significant increase in the peak intensity (PI) value of PCa (assessed using CEUS) with higher Gleason scores and MVD [11].

However, the prognostic value of MVD in PCa is contradictory. Its prognostic significance is still unclear. Some studies have found that MVD does not match the changes in tumor blood vessels after anti-angiogenic therapy for PCa because tumor angiogenesis is a complex process [12]. Tumor blood vessels require not only quantitative evaluation but also analysis of the morphology, structure, and function. Most of the new blood vessels in invasive PCa may be immature in morphology and function, lacking complete membrane and pericyte. The connections between endothelial cells may be loose. Tumor cells may be easier to penetrate, leak, and transfer because of the reduced structural barriers [13]. Therefore, MVD is not considered a useful parameter in the prediction of PCa prognosis [14]. Tumor microvessel maturity, which is defined by immature blood vessels, plays a critical role in the development of PCa [15]. Researchers observed a strong association between the size and shape of the microvessels and the incidence of bone metastases or cancer death in several years following diagnosis [16]. In some studies of anti-angiogenic therapy for PCa, especially castration-resistant PCa, efficacy was not significant. One of the reasons may be related to the complex angiogenesis mode and microvascular maturity of the tumor [17]. However, there is no definitive method for the measurement of the degree of microvessel maturity. Information about the relationships among contrast enhancement patterns, the PI values of quantitative parameters, microvessel maturity, and ISUP grades are limited. Therefore, the discovery of a suitable non-invasive imaging method, which can be used to assess both microvessel maturity and the ISUP grade, could be valuable in selecting targets for prostate biopsies. This knowledge could change biopsy strategies, leading to a more appropriate therapeutic strategy. Therefore, in the present study, we aimed to identify whether the contrast enhancement patterns and PI values of quantitative parameters can be used to non-invasively evaluate the microvessel maturity and the ISUP grade of PCa.

## 2 Materials and methods

### 2.1 Patients

In this retrospective study, we included 61 patients with prostatic adenocarcinoma, who did not undergo treatment previously. These patients were admitted to the Clinic of Urology from January 2019 to June 2020 with increased prostate-specific antigen (PSA) levels in serum. All tumors were diagnosed to be primary, without previous therapy. In this study, we excluded patients who underwent PCa-related therapies, including radiation therapy, chemotherapy, and androgen deprivation therapy. The local Ethics Committee approved this study (Protocol number: 2021KY-E-238), and all patients gave written informed consent before their enrollment in the study.

### 2.2 CE-TRUS imaging

All patients included in this study underwent CE-TRUS before prostate biopsy. The ultrasound equipment used was the LOGIQ E9 system (GE Healthcare, Milwaukee, WI, USA) with a transrectal probe operating at a frequency of 3–9 MHz. During CE-TRUS, 2.4 mL of SonoVue (Bracco, Milan, Italy) was administered intravenously as a rapid bolus injection, followed by a 5 mL saline flush. The acoustic power of the equipment was set at a mechanical index of 0.10. The contrast imaging plane was considered the transverse plane of the CE-TRUS abnormality. The results of the imaging examinations were saved in a DICOM format.

### 2.3 Image and data analyses

Prominent differences exist in the contrast enhancement of peripheral zone (PZ) lesions and that of transition zone lesions. The normal inner gland and coexisting benign prostate hyperplasia often appear to be hypervascular; therefore, regions of interest (ROIs) were drawn only on the PZ biopsy sites on CE-TRUS images. However, in the case of systematic biopsies, ROIs with diameters of approximately 5 mm were traced around the biopsy sites. Furthermore, ROIs were drawn as closely as possible to encompass the CE-TRUS abnormalities in the case of targeted biopsies. The CE-TRUS image analysis was performed individually by two sonographers with more than 10 years of work experience, who were blinded to all clinical and pathological information. If the conclusions obtained by these sonographers were inconsistent, a consistent conclusion would be reached after discussion. According to the enhancement patterns of prostate lesions, the patients in this study were divided into low- and high-enhancement groups. An abnormal imaging lesion was defined with reference to the surrounding normal prostate tissue or compared to the other side, with asymmetric or asynchronous enhancement areas. Based on the normal internal gland, if the enhancement level was similar to or higher than that of the internal gland, high enhancement was defined. However, an enhancement level lower than that of the internal gland was defined as low enhancement. In the case of uneven enhancement, the enhancement level was based on the enhancement in more than 50% of the lesion area. Time-intensity curve (TIC) analysis for each ROI was performed using the TIC analysis software of LOGIQ E9.

### 2.4 Prostate biopsy

Within 1 week after CE-TRUS, 2–3 targeted biopsy cores were taken from areas of abnormal CE-TRUS findings, and a systematic 12-core transrectal ultrasound-guided prostate biopsy was performed by a sonographer with more than 10 years of experience in the field. Core samples were extracted using an automatic biopsy gun (C. R. Bard, Covington, GA, USA) that triggers an 18 G needle with a core length of 25 cm. Specimens were marked based on the site of the biopsy.

### 2.5 Pathological analysis and immunostaining

Tissue samples from PCa patients were extracted using ultrasound-guided targeted or systematic biopsy. The grading of tumors on needle biopsy samples was undertaken in accordance with the ISUP grades. Each sample was assigned to one of the following ISUP grades: grade 1 (GS ≤ 3 + 3), grade 2 (GS 3 + 4), grade 3 (GS 4 + 3), grade 4 (GS 4 + 4, 3 + 5, and 5 + 3), and grade 5 (GS 9–10).

The biopsy specimens of prostate tissues were immersed in formalin for 5–6 h in preparation for optimal immunostaining and then these sample tissues were embedded in paraffin. A pathologist, with more than 10 years of experience in this field, reviewed all the histological slides from ultrasound-guided targeted or systematic biopsies and selected a few slides for quantitative evaluation. The selected samples were stained for CD31 using the “hot spot” method introduced by Weidner et al. [18] for the assessment of the MVD. After staining, the most vascularized areas were identified using a fluorescence microscope with a 10× objective, and three fields were selected for counting the vessels at 20× magnification. The average count was designated as the MVD. Without a vascular luminal structure, single vascular endothelial cell buds, strand-shaped endothelial cells, or clustered endothelial cells were considered as immature vessel. The vessels that showed an obvious luminal structure were considered relatively mature vessels. The calculations used for mature and immature vessel indices are as follows:

\text{Mature}\hspace{.25em}\text{vessel}\hspace{.25em}\text{index}=\text{Number}\hspace{.25em}\text{of}\hspace{.25em}\text{relatively}\hspace{.25em}\text{mature}\hspace{.25em}\text{vessels}/\text{MVD}]

and

\text{Immature}\hspace{.25em}\text{vessel}\hspace{.25em}\text{index}=\text{Number}\hspace{.25em}\text{of}\hspace{.25em}\text{immature}\hspace{.25em}\text{vessels}/\text{MVD}.]

Paraffin-embedded PCa tissue sections with a thickness of 4 μm were obtained. The sections were mounted on glass slides coated with silane. These sections then underwent deparaffinization using xylene and rehydration with graded alcohol solutions. The treated sections were pressed with citric acid. Next, they were rinsed with phosphate-buffered saline (PBS) and treated with 50 μL of 3% H_2_O_2_ solution for 10 min at room temperature for inactivation of endogenous peroxidase. Non-immune goat serum (50 μL) was added to each section for incubation at room temperature for 10 min. The sections were further incubated with CD31 antibody (MAB-0720) at room temperature for 60 min. After incubation with the primary antibody, the sections were washed with PBS three times. Then, the sections were again incubated with 50 μL of the immunochromogenic reagent D-3004-15 (secondary antibody, horseradish peroxidase [HRP] labeled) at room temperature for 30 min. After adding 100 μL of freshly prepared DAB chromogenic solution to the sections and washing them thrice, the sections were stained with DAB chromogenic solution (100 μL), incubated for 3–5 min, and photographed using an optical microscope (Olympus Corporation, Tokyo, Japan). The sections were rinsed with tap water, counterstained with hematoxylin, rinsed with PBS to return to blue, dehydrated with gradient alcohol, rendered transparent with xylene, and finally sealed with neutral gum. The cytoplasm of vascular endothelial cells of all study sections showed a strong positive reaction.

### 2.6 Statistical analysis

All analyses were conducted using SPSS 25.0 (SPSS Inc., Chicago, IL, USA). The comparisons between sample means of the two groups were performed using an independent sample *t*-test or non-parametric Mann–Whitney test. Correlations were analyzed using Pearson or Spearman correlation analysis. A statistically significant difference was indicated by *P* < 0.05.

## 3 Results

### 3.1 Patient characteristics

The mean age of the patients included in this study was 68.2 ± 9.5 years (range, 50–90 years). The median value of the pretreatment serum PSA was 74.45 (range, 5.12–1000.00 ng/mL). The final pathological staging for the 61 patients with PCa was recorded as 7, 6, 16, 13, and 19 cases with ISUP grades 1–5, respectively.

### 3.2 Differences in PI, MVD, microvessel maturity, and ISUP grades between PCa cases with different contrast enhancement levels on CE-TRUS

The number of mature vessels, MVD, mature vessel index, and ISUP grade were all higher in the high-enhancement group than in the low-enhancement group; however, the immature vessel index was lower in the high-enhancement group than in the low-enhancement group (Table 1).

**Table 1:** Comparison of PI, MVD, microvessel maturity, and ISUP grades between PCa cases with different contrast enhancement levels on CE-TRUS

	*n*	Mature vessels	Immature vessels	MVD	Mature vessel index	Immature vessel index	ISUP grade
Low enhancement	16	3.35 ± 1.91	15.08 ± 6.30	18.43 ± 6.46	0.19 ± 0.12	0.81 ± 0.12	3
High enhancement	45	6.18 ± 3.44	17.19 ± 6.26	23.37 ± 6.45	0.27 ± 0.14	0.73 ± 0.14	4
*t*/*z*		−3.118	−1.158	−2.631	−2.017	−2.017	−2.224
*P*		0.003	0.252	0.011	0.048	0.048	0.026

### 3.3 Correlations among PI, MVD, microvessel maturity, and ISUP grade in PCa

**Figure 1:**
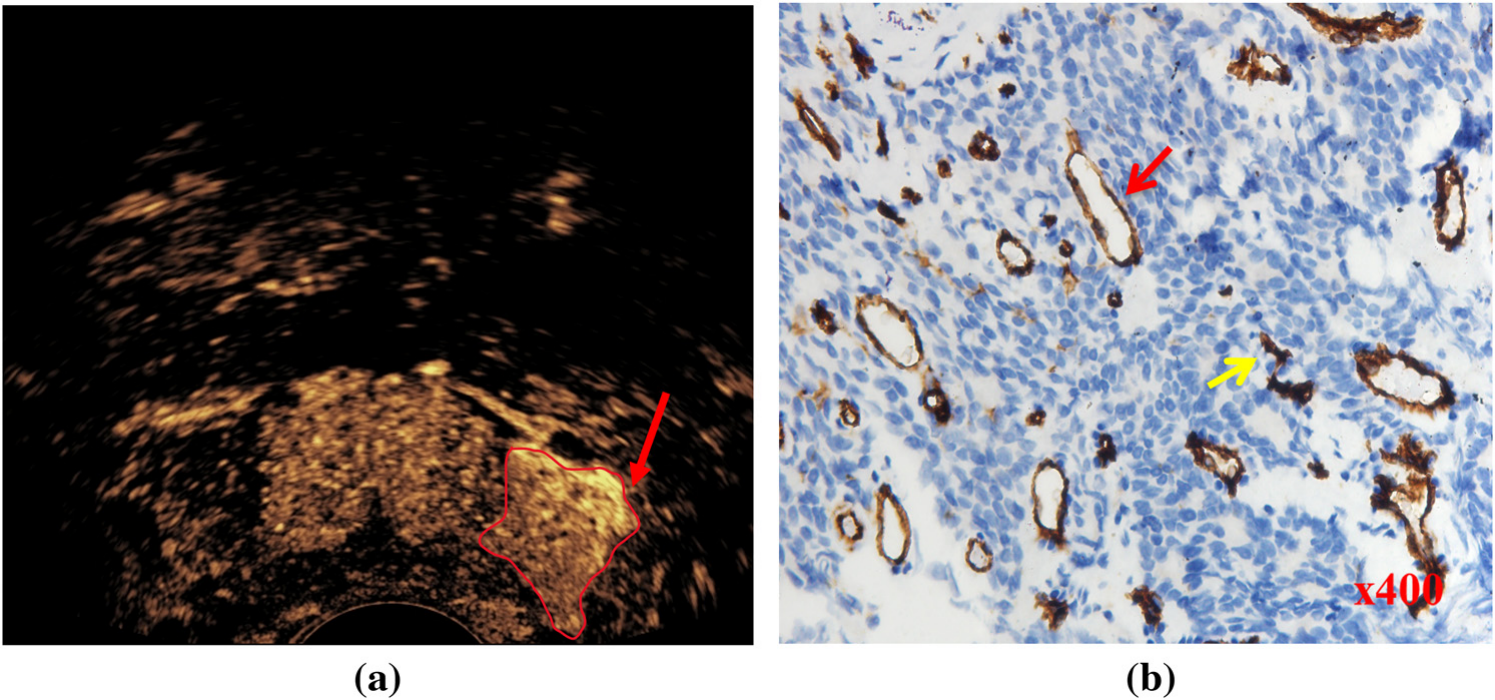
Representative images of CE-TRUS showing high enhancement levels and immunohistochemical staining. (a) The lesion in the left PZ of the prostate is shown with a high enhancement level on CE-TRUS (red arrow) with an ISUP grade of 5. (b) Targeted biopsy tissue stained for CD31 expression. Tumor cell nuclei (blue) are distributed in nests. Most dense groups of microvessels contain relatively mature vessels (red arrow), and the yellow arrow indicates an immature vessel.

**Figure 2:**
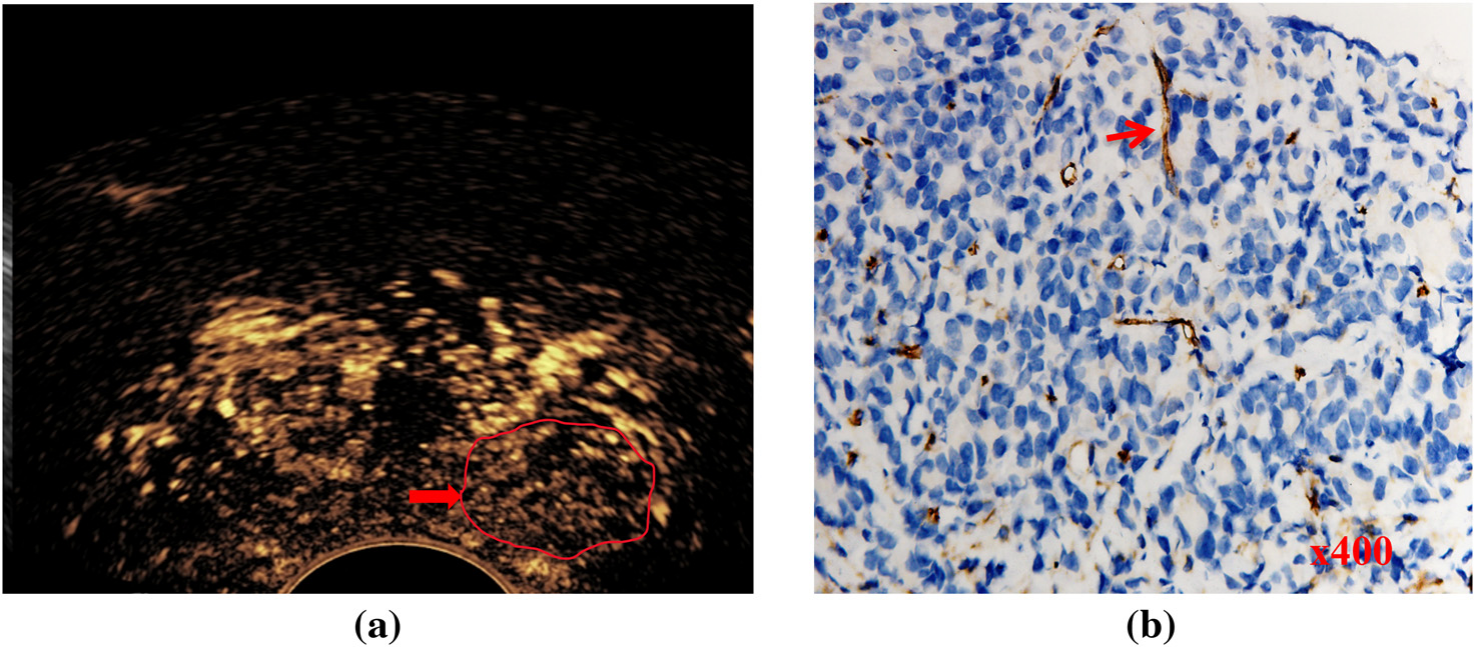
Representative images of CE-TRUS showing low enhancement levels and immunohistochemical staining. (a) The lesion in the left PZ of the prostate is shown with a low enhancement level on CE-TRUS (red arrow) with an ISUP grade of 4. (b) Targeted biopsy tissue stained for CD31 expression. Tumor cell nuclei (blue) are distributed individually throughout the tissue. Most dense areas of microvessels (brown–yellow) contain relatively immature vessels (red arrow).

The PI value was positively correlated with the number of mature vessels (Table 2, Figures 1 and 2). However, no correlations were observed among PI, MVD, number of immature vessels, mature vessel index, immature vessel index, and ISUP grades.

**Table 2:** Correlations among PI, MVD, microvessel maturity, and ISUP grades in PCa

	PI	
	*R*	*P*
Mature vessels	0.340	0.007
Immature vessels	0.081	0.536
MVD	0.220	0.089
Mature vessel index	0.170	0.189
Immature vessel index	−0.170	0.189
ISUP grade	0.234	0.069

### 3.4 Correlations among ISUP grades, microvessel maturity, and MVD in PCa

**Figure 3:**
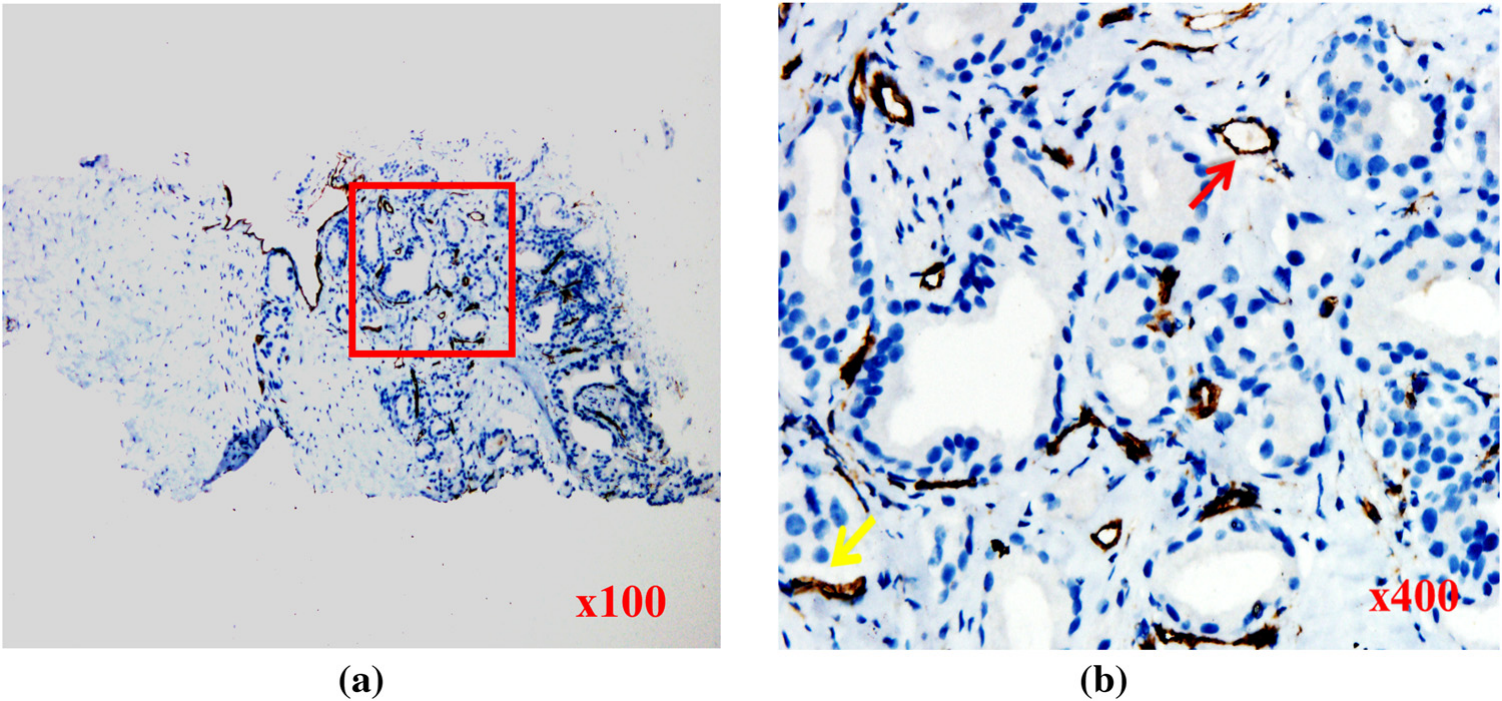
Targeted biopsy tissues from the PCa case with ISUP grade of 2, stained for CD31. (a) Abundant microvessels in tumor tissues (red box). (b) Tumor cell nuclei (blue) were small and poorly differentiated. The numbers of mature vessels (red arrow) and immature vessels (yellow arrow) were almost equal.

**Figure 4:**
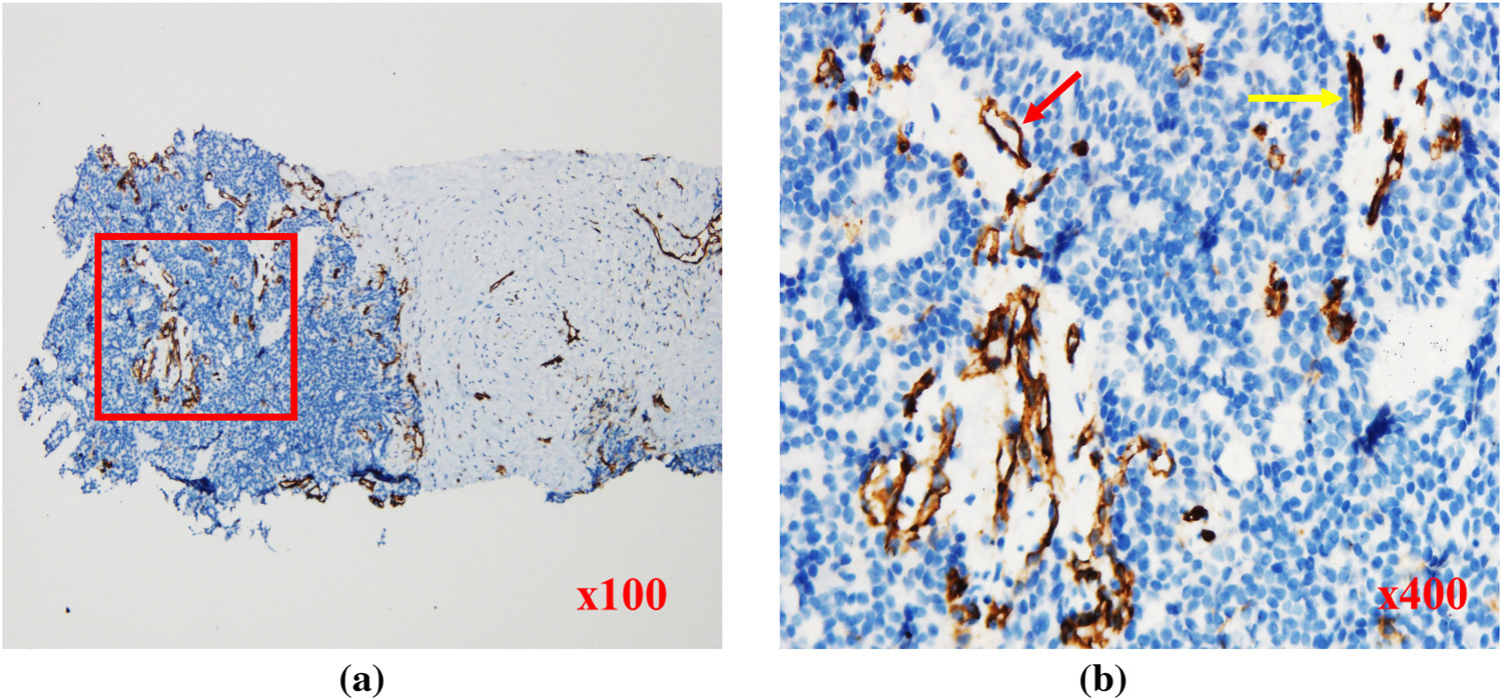
Targeted biopsy tissues from the PCa case with ISUP grade of 5, stained for CD31. (a) Abundant microvessels in tumor tissues (red box). (b) Tumor cell nuclei (blue) were densely distributed in flakes. Most were immature vessels (yellow arrow), with only a few mature vessels (red arrow).

The ISUP grade of PCa specimens was positively correlated with the number of immature vessels and MVD (Table 3, Figures 3 and 4). However, no correlations were found among ISUP grade, number of mature vessels, mature vessel index, and immature vessel index.

**Table 3:** Correlations among ISUP grade, microvessel maturity, and MVD in PCa

	ISUP grade	
	*R*	*P*
Mature vessels	−0.005	0.967
Immature vessels	0.465	0.000
MVD	0.417	0.001
Mature vessel index	−0.233	0.071
Immature vessel index	0.223	0.071

## 4 Discussion

ISUP grades can accurately predict the aggressiveness and prognosis of PCa [19]. Moreover, angiogenesis plays an important role in the development of PCa [20]. The complex angiogenesis mode and microvascular maturity of the tumor play a critical role in the development of PCa [15,17]. Therefore, a non-invasive imaging technique that can assess both microvessel maturity and ISUP grade could support timely diagnosis and determination of PCa characteristics.

Our results showed that MVD values in PCa cases with high enhancement on CE-TRUS were higher than those in PCa cases with low enhancement. The differences in the enhancement patterns on CE-TRUS images in PCa patients may be mainly due to the different proportions of relative mature vessels in these cases. When the mature vessel index is low, the perfusion of the lesion is poor. Although prostate tumors have abundant microvessels, their contrast enhancement may be at a low level due to the low number of functional mature vessels [21]. Therefore, the immature vessel index in the high-enhancement group was lower than that in the low-enhancement group, whereas the mature vessel index in the high-enhancement group was higher than that in the low-enhancement group. These results suggest that the enhancement pattern on CE-TRUS can reflect microvessel maturity in PCa patients.

Our results also showed that ISUP grading was higher in the high-enhancement group than in the low-enhancement group. A higher ISUP grade indicates worse tumor differentiation and a greater number of immature vessels in PCa. Theoretically, MVD should be higher than the number of immature vessels in the high-enhancement group. However, we obtained a conflicting result in this case.

In the present study, we analyzed the correlations among PI, MVD, microvessel maturity, and ISUP grade. A positive correlation was observed between PI and the number of mature vessels but the correlation was weak (*r* = 0.340). However, no correlations were observed between PI and MVD or ISUP grade. A possible explanation for such a scenario might be that the PI value we calculated was the average of all the pixels of the ROI at the peak time, but the signals induced by immature and mature vessels in the same ROI could affect each other. Therefore, the PI value that we obtained cannot represent the signal induced by individual immature or mature vessels. Second, the hemodynamics of a tumor is affected by cell proliferation, angiogenesis, and vascular maturity [22]. In this study, the vascular factors may be complicated, which could affect the distribution and metabolism of contrast agents, due to the heterogeneity of the structure and function of the tumor vessels [23]. The contrast parameters might change due to the differences in the number, diameter, and atypia index of microvessels at the edge and center of a lesion. In addition, the increase in vascular permeability, blood viscosity, and blood flow resistance within a tumor might also affect the metabolism of contrast agents [23]. Although the blood supply within the tumor is mainly dependent on the function of vessels, it is also affected by complex factors, such as tumor necrosis, hemorrhage, and fibrosis. Studies have shown that inflammation is a cause of PCa, which may play a crucial role in the occurrence and progression of PCa, including initiation, promotion, malignant transformation, invasion, and metastasis [24]. Some pro-inflammatory cytokines, such as IL-17, may affect several landmark functions of PCa occurrence and development, such as inhibition of proliferation, resistance to cell death, activation invasion, and induction of angiogenesis [25]. In the meantime, each Gleason score is the result of the sum of essentially heterogeneous categories (Gleason patterns [GP]), as each pattern contains several tumor forms. Previous research has shown that the patterns of GP5 and tumor necrosis are associated with poor histopathological characteristics and high residual disease rates [26]. Therefore, simply using PI values to assess the function and status of tumor neovascularization is not accurate.

An important limitation of our study was the limited pathological evaluation due to a lack of correlation of results with prostatectomy. Previous studies reported undergrading of prostatectomy Gleason scores in biopsy samples [27]. Second, the maturity of microvessels needs to be evaluated based on certain indicators, such as size and regularity, which could be considered in future studies. Third, this study had a small sample size. Therefore, further studies with large sample sizes could provide more generalizable results.

## 5 Conclusion

We conclude that the enhancement pattern on CE-TRUS could reflect the microvessel maturity in PCa tumors. The immature vessel index in the high-enhancement group was lower than that in the low-enhancement group, whereas the mature vessel index in the high-enhancement group was higher than that in the low-enhancement group. ISUP grading was higher in the high-enhancement group than in the low-enhancement group. The PI value was positively correlated with the number of mature vessels.
